# Trumping the Centers for Disease Control: A Case Comparison of the CDC’s Response to COVID-19, H1N1, and Ebola

**DOI:** 10.1177/00953997221112308

**Published:** 2023-01

**Authors:** Eleanor Schiff, Daniel J. Mallinson

**Affiliations:** 1 Penn State University, University Park, PA, USA; 2 Penn State Harrisburg, Middletown, PA, USA

**Keywords:** bureaucratic failure, bureaucratic control, case comparison, COVID-19, public health

## Abstract

Despite being the richest and most prepared nation in the world, the U.S. responded badly to the COVID-19 crisis. This paper examines the nature of political control and the essence of bureaucratic failure for the Centers for Disease Control and Prevention (CDC), an independent agency. In three case studies, we analyze the CDC’s success in handling H1N1 and Ebola, and its failures on COVID-19. We find that the CDC suffered not only from political interference by the Trump Administration but also internal organizational problems that muted its ability to respond effectively. We conclude by offering policy prescriptions for addressing concerns of bureaucratic autonomy and success at the CDC.

The COVID-19 pandemic has been a disruptive force worldwide; however, some countries have fared better than others in managing the disease. The U.S., the richest country in the world, horribly mismanaged the crisis, leading to over 700,000 deaths as of October 2021. Prior to the COVID-19 crisis, bio-security researchers ranked the U.S. the most prepared for a pandemic (Maxmen & Tollefson, 2020). At almost every important moment in the first 6 months of 2020, however, the U.S.’ response to COVID-19 was a dossier of disaster.

During the Trump Administration, the American response was wholly underwhelming and inadequate in controlling the virus, much to the bafflement of Americans and the rest of the world. Poor countries, such as Vietnam and Uganda, were, at least initially, more successful at controlling the virus than the U.S., and rich countries like Taiwan quickly controlled the virus’ spread and experienced few deaths. Why was the U.S. in this position? Why did the Centers for Disease Control and Prevention (CDC), one of the most admired American government agencies across the world, largely fail in providing adequate guidance for COVID-19 when they successfully handled past scares such as Ebola (2014) and H1N1 (2009)? In this case comparison study, we will compare CDC’s largely failed response to COVID-19 to its strong leadership during both the Ebola and H1N1 outbreaks. We argue that CDC’s shortcomings in adequately handling COVID-19 are beyond the feeble leadership of President Trump (NEJM Editors, 2020). They stem from both bureaucratic intransigence and political interference.

This article begins with a brief background on the CDC as an institution, including its typically stellar reputation in infectious disease control. We then draw from the literatures on bureaucratic political control and bureaucratic failure to examine how well the CDC is politically controlled. We then compare the cases of H1N1, Ebola, and COVID-19 to examine the different outcomes in similar situations. Finally, we present conclusions and offer potential policy prescriptions to overcome the CDC’s current shortcomings.

## Brief Background on the CDC

**Figure 1.**
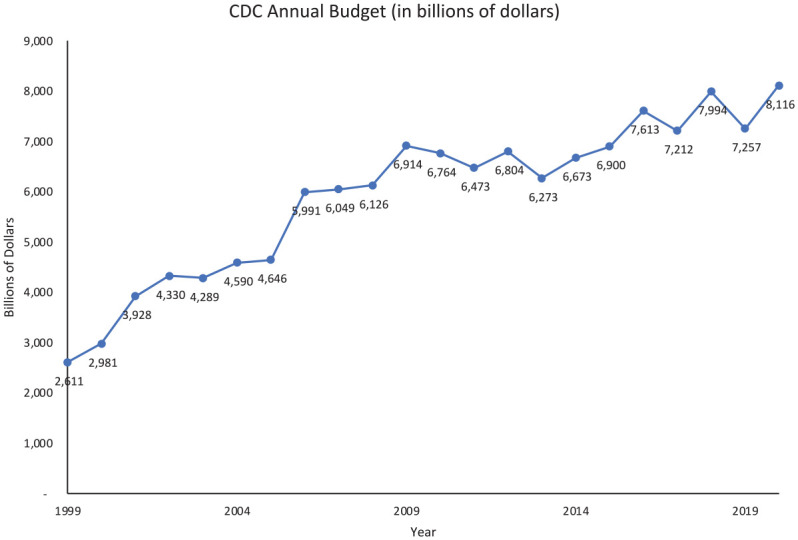
CDC’s annual appropriation 1999 to 2020.

The CDC was founded in 1946 and headquartered in Atlanta to lead efforts eradicating malaria in the American South. The agency has enjoyed widespread public and Congressional support and confidence in the U.S. and across the world for its scientific expertise and advice (Interlandi, 2021). It offers guidance to states, physicians, researchers, and government officials on a variety of topics from vaccine schedules for kids to contagious disease mitigation. Its mission is to work “24/7 to protect America from health, safety and security threats, both foreign and in the U.S.” (CDC, 2019b). Historically, CDC has been well funded and in fiscal year 2020, its combined budget was $8.116 billion (Figure 1). Organizationally, CDC is divided into five separate divisions: Occupational Safety and Health, Public Health Service and Implementation Science, Public Health Science and Surveillance, Noninfectious Diseases, and Infectious Diseases.

The COVID-19 pandemic has been the worst public health crisis in the U.S. since the Spanish Flu pandemic of 1918 and has challenged CDC significantly. Chronic underinvestment in the nation’s public health infrastructure at the local, state, and federal levels has exacerbated this problem despite warnings that a pandemic was not only possible, but probable in the future (Maani & Galea, 2020; Xu & Basu, 2020). In fact, the COVID-19 outbreak followed the predictions of prior preparedness exercises, yet the U.S. government was not able to mitigate its spread (Maxmen & Tollefson, 2020). Why has the U.S. struggled to effectively coordinate its federal COVID-19 response, despite prior planning and CDC’s stellar reputation? Why did the CDC perform effectively with prior pandemic scares, yet struggled during the COVID-19 pandemic? We argue that the CDC was not only plagued by political interference from the Trump White House, but that the COVID-19 pandemic exposed underlying, internal bureaucratic failures of intransigence and inflexibility. Prior to examining our three case studies, we turn to reviewing the literature on political control of bureaucracy to develop this argument.

## Political Control of the CDC

Political control of the CDC is analyzed through the lens of how the president and the Congress jointly provide policy direction. In a classic principal-agent paradigm, both institutions direct their agents in the federal bureaucracy to carry out their policy directives with fidelity (Miller, 2005). If a civil servant acts in a manner incongruous to the principals’ intention, agency loss occurs (Kiewiet & McCubbins, 1991). Not surprisingly, agency loss is a perennial problem in government, especially when political masters direct civil servants to implement policy that may be at odds with their own personal perspective (Brehm & Gates, 1997). In agencies such as the CDC, there are very few political appointees, and the agency operates as an autonomous unit letting science dictate its actions.

The organization, composition, and responsibilities of agencies shape the degree of political control exerted by principals (Schiff, 2020). Arguably, in relatively independent agencies such as the CDC there is less of a principal-agent problem between political and career civil servants since the medical professionals at the agency are guided by the standards of their profession rather than the political winds of Washington. Indeed, until COVID-19, the CDC was viewed as the world’s “premier public health agency” (McCay, 2020) operating in a non-partisan and scientific-based manner. Political interference on scientific guidance during Ebola, Zika, and H1N1 was absent. Unlike other agencies, such as the Office of Management and Budget, where political concerns and fidelity to the president’s agenda are of greater importance, CDC was widely seen as being above politics and advising the country on best practices. Given that the CDC is relatively isolated from politics, as it is headquartered in Atlanta, the theoretical expectations for how it might be politically controlled by its de-facto principals, the president and Congress, are different.

### A President’s Prerogative

The president has direct oversight responsibilities for all executive branch agencies. Presidential control of the bureaucracy is exercised through three basic mechanisms: appointment power, agency reorganization, and a first-mover advantage in agency budgeting (Meier, 1980; Moe, 1984; Wood, 1988; Wood & Waterman, 1991). A president nominates and appoints all leadership in Cabinet departments such as the Secretary, Deputy Secretary, and political appointees. A president will appoint upwards of 4,000 people who then oversee and manage career civil servants to execute the work of government. Political appointees owe their continued employment to the president who can remove them without cause at any time (with a few exceptions). Therefore, fidelity to a president’s agenda is expected and executed by political appointees since they serve at the president’s pleasure. Appointment power is one of the most important tools a president has to control agencies (Lewis, 2010).

The CDC is different, however, in that it is geographically distant from the President, with its headquarters in Atlanta, GA, not Washington, DC. The small handful of political appointees—6 out of the 11,000 people who work there (Florko, 2020)—are typically medical professionals who also hold a loyalty to the standards of their profession and Hippocratic oath. How well then, can a president expect to exert political control over the CDC? In our view, not well. First, there are very few political appointees at the agency, and it has a culture of “avoiding politics.” (Florko, 2020). Second, most of the work done and guidance issued is *not* performed by political appointees, but by staff scientists and medical professionals. Third, the CDC is not physically close to the Department of Health and Human Services (DHHS), its parent agency, or the White House, which limits close monitoring. CDC is thus well insulated from direct presidential interference.

Reorganization is a second potent tool of executive political control (Meier, 1980). Clarifying reporting responsibilities and solidifying hierarchical structures is one mechanism that the president can use to assert greater control over agencies. The classic example of agency reorganization is President Kennedy’s establishment of the Peace Corps. Congress was hostile to the program as a potential haven for Vietnam War draft dodgers and, many in Congress shared the attitude that the U.S. should not send energetic young people to developing countries when there were plenty of problems in the U.S. that deserved equal attention (Howell, 2005). Kennedy, in defiance of Congress, issued Executive Order 10924 establishing the Peace Corps within the State Department and dispatched volunteers worldwide. When Congress finally approved of the program, it was a *fait accompli* since it was already popular domestically and abroad, and Congress had no choice but to sanction its creation.

For CDC, however, reorganization is less of a threat since it is a well-established agency and trusted globally. In 2004, President George W. Bush proposed reorganizing the National Institute for Occupational Safety and Health (NIOSH) and lowering its importance in the CDC hierarchy; however, the change in the status quo was met with swift and vehement opposition from scientists, unions, and industry leaders, forcing Bush to suspend the reorganization (Laborers’ Health & Safety Fund, 2005).

Other presidents have also tried to partially reorganize how the CDC conducts its business. In June of 2019, President Trump issued Executive Order 13875 “Evaluating and Improving the Utility of Federal Advisory Committees,” which disbanded advisory committees across the executive branch, including the CDC. At the CDC there had been several committees comprised of professionals throughout the field that advised the director and others on various topics about public health. While Trump disbanded these committees, this was not a true re-organization of the CDC. He asserted that the committees had fulfilled their original mandate and were no longer needed. The EO received no mention from the mainstream media such as *The New York Times* or *The Wall Street Journal* and generally received no press. Therefore, this second tool to exert Presidential control over the CDC is a hollow lever.

The final mechanism for a president to exert control over executive agencies is one shared with the Congress: the agency’s budget. The president has the first-mover advantage in drafting agencies budgets. Delegated by Congress through the Budget Act of 1921, the president is required to submit to Congress a draft budget for the entire government by the first week of February. This enables the president to propose a budget for an agency and Congress is left to react to it. This is yet another empty attempt at control for the CDC, as it is largely regarded as an apolitical agency, and it has enjoyed bipartisan support across decades. While there are annual ebbs and flows in its budget, generally funding has increased. Looking at Figure 1, CDC’s budget increased 68% from 1999 to 2020. The Trump administration also proposed to increase its budget even prior to the COVID-19 crisis.

### Congressional Dominance

Congress is the other constitutional master of executive branch agencies and, through statute, directs them to carry out its will in drafting regulations and implementing programs. According to the Congressional Dominance theory, there are three basic mechanisms for how Congress can assure bureaucratic adherence to their will: *ex ante* statutory control, *ex post* monitoring, and budgeting (McCubbins & Schwartz, 1984; Moe, 1987; Ringquist et al., 2003).

Congress’s committee system is the central organizational unit ensuring accountability for any bureaucratic agency, including the CDC. Through hearings, lawmakers can communicate directly with agency leadership to articulate and clarify the committee’s intent and the sense of Congress for agency activity (Weingast & Moran, 1983). The committee can ensure agency fidelity through *ex ante* (before the fact) statutory control and write legislation that is either very specific directing how agencies should implement programs, effectively constraining an agency, or very vague giving an agency more flexibility (Huber & Shipan, 2002).

Alternatively, Congressional committees also employ *ex post* (after the fact) monitoring. This is a more passive form of supervision yet more efficient for lawmakers in terms of their time, cognitive capacity, and electoral prospects. By allowing a third party to pull the proverbial “fire alarm,” the committee can sanction an agency as a problem occurs and benefit at the ballot box by resolving issues for a constituent (McCubbins & Schwartz, 1984). Unless they are alerted to a problem, the committee assumes that the agency is fulfilling its responsibilities.

Cutting, or threatening to cut, an agency’s budget is a powerful tool for enforcing Congressional will. If an agency does not implement a committee’s policy preferences with fidelity, its budget can be cut. Of course, if the agency is responsive, their budget can be increased the following year. Though this is a shared power with the president, Congress ultimately holds the power of the purse and has a stronger influence over domestic policy (Canes-Wrone et al., 2008).

In terms of controlling the CDC, Congress, for the most part, has taken a passive role in providing active oversight of the agency (Interlandi, 2021). The CDC has enjoyed a flush budget across administrations and changes in political control of the Congress (see Figure 1). This implies either that the CDC is well controlled by Congress, since it continues to be rewarded with bigger budgets, or that the Congress is passively enabling it to perform its job to protect the American public.

### Bureaucratic Autonomy

In examining both presidential and Congressional mechanisms to control the CDC, the political masters have largely acted as generous patrons enabling agency experts to fulfill their mission outside of strict political supervision. The CDC has very few political appointees among its large workforce, and both the political appointees and the career civil servants are in the mold of Weberian bureaucrats (Weber, 1978). They are experts in their field—as many of the CDC’s employees are either medical doctors or hold a PhD in a scientific field—and implement policy impartially to serve the greater good. Bureaucratic accountability in this context connotes fidelity to standards of the profession where they are less guided by political directives but more by peer-to-peer monitoring (Brehm & Gates, 1997). Weberian bureaucrats, along with other external conditions give rise to bureaucratic autonomy.

Agencies that are basically independent, such as the CDC, can work outside of their direct reporting structure and exercise independent policymaking power (MacDonald, 2015). In defining bureaucratic autonomy, Carpenter (2001) writes that it “occur[s] when bureaucrats take actions consistent with their own wishes, actions to which politicians and organized interests defer even though they would prefer that other actions (or no action at all) be taken” (p. 4). This can occur when three factors are simultaneously realized. First, autonomous agencies must be politically differentiated from the political bodies that work to influence and control them. The CDC is not located in Washington, D.C. and its proximity to the capital affects both presidential and Congressional influence (Kennedy, 2015; Selin, 2015) and is a distinct part of DHHS due to its own mission and history. Second, an agency must develop unique organizational capacity. Clearly the CDC fulfills this quality. It is respected worldwide for its expertise on diseases and public health guidance. Third, an agency must have political legitimacy. This implies that the public and lawmakers respect their expertise. There are multiple networks of professionals that the CDC can activate to fulfill its goals or protect its interests, if needed. Further, the agency can leverage high public approval as a resource to maintain its autonomy (Pew, 2020).

CDC clearly meets all three criteria of bureaucratic autonomy. This enables it to operate outside political micromanagement from the president and the Congress. In addition to the criteria for autonomy outlined above, agencies exercise autonomy when they respond to the needs of interest groups without being captured by them (Fukuyama, 2014). At least prior to COVID-19, the CDC operated in this realm of bureaucratic autonomy. Additionally, the CDC, like the Food and Drug Administration (FDA), has curated and cultivated a stellar reputation not only in the United States but also across the globe for employing neutral competence to public health problems (Carpenter, 2010). Multiple organizations such as state public health departments, physician groups, the U.S. Congress, and the American public rely on the CDC to provide premier public health guidance. Its reputation as the premier public health organization in the world engenders administrative discretion for the scientific employees who work there and therefore bureaucratic autonomy (Busuioc & Lodge, 2016; Carpenter & Krause, 2012) particularly during a novel crisis such as COVID-19 (Maor, 2010; Maor et al., 2013). We argue that the COVID-19 crisis led to erosion in the CDC’s autonomy, legitimacy, and public confidence. Political interference from the Trump administration exacerbated the COVID-19 crisis and left the CDC handicapped to provide clear and consistent guidance to the American public about how best to control and contain the virus. Further, bureaucratic intransigence internal to the CDC also contributed to its inability to effectively manage the COVID-19 crisis at the beginning of the pandemic. In contrast to political control, bureaucratic failure, detailed below, is a result of *internal* institutional failures.

### Bureaucratic Failure

Unlike the rich literature on both market failure and principal-agent theory, there is no broad theoretical perspective on the causes and consequences of government failure (Weimer & Vining, 2017). The scholarly work available has largely been developed by public choice scholars who have focused on bureaucratic failure as a failure of efficiency. The need for government to intervene is often caused by market failure, or the absence of a private market to produce a good—such as infectious disease control. Government is a non-profit monopoly that does not face the typical market pressures from competition that produce efficiency (Simmons & Tullock, 2011). Since bureaucratic agencies have no private sector competitor for the outputs they produce, evaluation of an agency’s success, or failure, has mostly been focused on economic efficiency.

Since government is not subject to the market controls of marrying supply based on the demand in the marketplace (Grand, 1991), many public choice scholars have concluded that government failure is a type of economic failure leading to inflated budgets and perverted cost functions (Niskanen, 1968). Government agencies do not have to pass a “market” test or face market pressures to survive annually, leading to “discretionary budgets,” which is the difference between the budget that the agency receives and the actual cost the agency incurs to produce a particular public good. Members of Congress have a difficult time knowing an agency’s cost function due to principal-agent problems including informational asymmetries, agency loss, hidden information, and the like.^1^ From this perspective, bureaucratic failure is a failure of using resources wisely and tax dollars efficaciously.

From the public choice perspective, the CDC meets the classic non-market controls other agencies face. It is a monopoly provider of guidance about public health to the 50 state departments of health, and it also is consulted internationally for its expertise. It would meet the definition of government failure in terms of economic efficiency as the supply of and demand for its services are not governed by competition, leading to potentially bloated budgets and politicians’ inability to understand the cost function of the services it is providing. Additionally, Congress and the President face principal-agent problems with monitoring the CDC’s scientific and medical advice.

Economic theory is certainly one perspective, with a rich theoretical tradition, but bureaucratic failure (or success) can also be based on the *perceived* effectiveness of an agency (Wolf, 1993). Having a strong institutional mission enables civil servants to develop an esprit de corps and work together on a shared mission (Fukuyama, 2014). The CDC has historically been a highly effective agency. It enjoys autonomy, strong leadership from health care professionals, a distinct mission and commitment to public health, a long history of serving the public, and bipartisan political support.

Thus, the CDC has all the institutional characteristics of an agency that is highly effective and autonomous. Why then, did it experience a failure during the novel COVID-19 pandemic? We offer three explanations. First, the Trump Administration eroded the CDC’s autonomy. It politicized decision-making, and muzzled CDC’s leadership in providing scientific guidance to the public on appropriate mitigation and safety measures for the virus. Second, the CDC suffered from institutional rigidity (Fukuyama, 2014) in its inflexibility in working closely with the FDA to utilize the capacity of universities in the U.S. to process tests at the beginning of the pandemic. It also botched the new test for COVID-19 at the beginning of the pandemic. As an institution, it was unable to adapt quickly to the speed of the pandemic and overcome bureaucratic red tape to stop the spread of the virus. Lastly, COVID-19 was a novel virus and the CDC lacked both the institutional capacity and mission to coordinate across the federal government. We turn now to evaluating the CDC’s response to COVID-19 by contrasting it with the H1N1 global pandemic in 2009 and the regional Ebola epidemic in 2014. A case comparison methodology will allow us to distinguish the effects of political interference during COVID-19 from longer-standing issues of institutional rigidity that affected responses to prior disease outbreaks.

## CDC’S Autonomy During Three Crises: A Case Comparison

There are many approaches to case comparisons (Druckman, 2005). We took the approach of selecting cases that offer many similarities, but that had distinct differences in outcomes. We chose to look only at cases with respect to the CDC, not comparing to other countries, because the goal is to understand why it experienced a high degree of failure during one case, but not the others. For our case selection, we chose the H1N1 outbreak in 2009, Ebola in 2014, and COVID-19 in 2020. H1N1 and Ebola were chosen because in both the CDC was responding to a virus outbreak emerging from outside the United States. Ebola is the most different, as it did not spread in the U.S., but it was highly feared. Also, all three cases were subject to disinformation being spread largely online. Granted, online communications evolved substantially between 2009 and 2020, but each captures CDC’s efforts to adapt to new communications environments. Finally, we selected these three cases because public opinion data with consistent question wording is available for the studied period. We chose not to examine CDC’s failed response to the early AIDS epidemic because of the substantially different morality framing of the disease.

### H1N1

While the exact origins of H1N1, colloquially known as “swine flu,” are disputed, it was a novel flu-like virus first reported in April of 2009 (CDC, 2019a; Wenzel, 2010). Unlike COVID, or SARS in 2003, this flu strain began in North America and predominately affected people younger than 60 (CDC, 2019a). To contain the impact of the virus and alert countries worldwide about the novel threat, the CDC moved quickly to report new infections and the World Health Organization (WHO) declared it a global pandemic in June of 2009. Officially, the pandemic lasted from 2009 to 2010, after which it gradually disappeared. The H1N1 strain was a more infectious and more severe strain of the seasonal flu and an estimated 284,000 people died in 2009 of respiratory or cardiovascular complications associated with H1N1 (Dawood et al., 2012) as compared to 36,000 during a regular flu season (USA Facts, 2021).

The CDC’s director, Dr. Thomas R. Friedman, marshalled the agency to declare a public health emergency in the U.S. at the end of April 2009, shortly after cases were confirmed in California and then later Texas. The CDC’s laboratory was the first entity in the world to identify the new flu strain in April 2009, and it uploaded the complete gene sequencing of the H1N1 virus to a publicly available international database for scientists around the world (CDC, 2019a). Under Friedman’s leadership, the CDC issued guidance documents to the public, businesses, clinicians, laboratories, and schools on how to remain open safely. Additionally, the CDC coordinated with the FDA to get diagnostic testing approved on an emergency use basis at the end of April 2009, just weeks after the new virus was detected, and began shipping the test kits to state public health departments and public health officials worldwide (442 labs in 145 countries; CDC, 2010). Further, the CDC worked quickly to develop a vaccine in “near record time” that was deployed in the fall of 2009 (CDC, 2010). The CDC also coordinated a vaccination outreach campaign. President Obama added the prestige and the weight of the White House in declaring January 10 to 16, 2010, the National Influenza Vaccination Week in order to prod the public into getting vaccinated.

In the CDC’s handling of the H1N1 pandemic, it acted quickly and authoritatively in handling the threat to public health. It provided clear and sound guidance to keep people safe in working to slow the spread of the disease. Schools remained open throughout the crisis, and public health officials at the CDC responded in the predictable ways that the public expects of its professionals. H1N1 also did not become a political issue—and there was no political interreference from the White House or others in Congress, unlike COVID-19. The CDC also had to contend with misinformation online and leverage new tools, like YouTube, to communicate with the public (Pandey et al., 2010; Walton et al., 2012). In short, the CDC responded to the H1N1 crisis in the manner the public would hope and expect of such a prestigious and venerated public health organization. It performed with excellence and sustained the public’s confidence.

### Ebola

Ebola is a rare, but highly fatal, disease that emerged in central Africa in 1976. In 2014, a new outbreak of Ebola in western Africa proved difficult to control and sparked fears of global transmission. The first case was identified on March 23 in Guinea and the disease rapidly spread with little control to urban areas in that country, Liberia, and Sierra Leone. On August 8, the WHO declared the Ebola outbreak a “Public Health Emergency of International Concern.” As the epidemic worsened in western Africa, healthcare systems in affected areas collapsed and further hindered the response (Bell et al., 2016). Working with the WHO and other international partners, the CDC instituted it’s “largest emergency response in the agency’s history” (Bell et al., 2016, p. 7). CDC deployed a total of 1,897 staff to affected countries, established an incident management system, and established information gathering, analysis, and reporting systems to improve the public health response. CDC also developed its own case modeling in September 2014. On the home front, CDC was active in preparing for rapid testing and establishing a three-tiered system for American healthcare facilities: (1) frontline; (2) Ebola assessment; and (3) Ebola treatment. CDC also worked with Customs and Border Patrol and the Department of Homeland Security to screen and track travelers from West Africa, including the agency’s first ever use of fever monitoring that would become ubiquitous during the COVID-19 pandemic (Bell et al., 2016; Gostin et al., 2014).

CDC also faced communications challenges that would rear their heads again during the COVID-19 pandemic. In terms of traditional media, the agency faced the filtering of its authoritative voice by TV journalists (Kott & Limaye, 2016). Some outlets would directly convey the messages of CDC scientists, but primetime coverage from outlets on the right, left, and center tended to use non-CDC government officials, academics, and medical experts instead of CDC experts to offer commentary on the disease. Kott and Limaye (2016) point out that the agency faced a multi-level translation of its messaging. For example, President Obama would speak about Ebola and his characterization may be presented on a show such as the O’Reilly Factor (Fox News), but host Bill O’Reilly would then offer his own commentary. O’Reilly shifted the framing from medicine to socio-politics. The CDC’s long press releases and statements would have to be repackaged into smaller bites by all press outlets and were often attached to the type of storytelling valued by journalists and viewers. Not being savvy to this process can hinder crisis communications about an infectious disease outbreak (Kott & Limaye, 2016).

Further, CDC had to contend with social media in a way that would become even more central by 2020. The agency was active in using social media to provide information to the public and dispel misinformation about the disease’s etiology (Carter, 2014; Lazard et al., 2015). Analyses of Tweets from the agency and public showed that the public wanted information about the virus before the problem became a crisis in the U.S. and that “the CDC is viewed primarily as a credible source of information” (Crook et al., 2016, p. 353). The CDC’s Twitter followers ballooned in 2014, and the agency “saw social media as a way for the CDC to emerge as the source for ‘credible, fact-based’ information about the outbreak” (Dalrymple et al., 2016, p. 443).

CDC’s messaging during the Ebola outbreak has been criticized however, Its guidance on quarantining healthcare workers returning from western Africa has been critiqued as not based on science and having had a chilling effect on workers wanting to assist in the crisis response. Gatter (2014) argues that the guidance was a response to public fears that were exacerbated by CDC’s own missteps. Early on, then CDC director Tom Frieden made statements that hospitals were ready. Those statements were followed by the poor handling of several cases of healthcare workers returning from western Africa and a Liberian man vising his family in Texas. Emblematic of the failure was the CDC’s allowance of a nurse to fly on a commercial flight *after* she informed the agency that she had been exposed to an Ebola patient and had a low-grade fever (Gatter, 2014). In addition to consequent bipartisan backlash in Congress, states began instituting their own quarantine rules. Most famously this led to a standoff between the Obama Administration (which was opposed to the rules), nurse Kaci Hickox, and Governor Chris Christie (R-NJ). Ultimately CDC responded with stricter quarantine rules that went beyond prevailing Ebola science.

The case of Ebola was largely a success for the CDC, given that it was the largest agency response to an infectious disease to date. However, cracks in effective communication and the challenges of rising social media were prescient for COVID-19. CDC did not face the political interference that is apparent in the COVID-19 case, but overstepping Ebola science in setting quarantining rules was driven by political concerns and growing public fear. One final distinction for Ebola is notable. When the disease emerged in 2014, it was not novel. CDC had information on the etiology of the disease and how to treat it and could act quickly to transmit this information. This is not the case for COVID-19.

### COVID-19

It is still debated exactly when and how COVID-19 emerged. Signs now point to the late fall of 2019, but its rapid global spread is indisputable. Also indisputable is the unprecedented political interference from the Trump Administration in the agency’s response to the pandemic. Though, political interference could be described as creeping, not immediate (Weiland, 2020). CDC Director Robert Redfield reported the COVID-19 threat to HHS Secretary Alex Azar on January 3, but Azar waited two full weeks before briefing President Trump (Ballhaus & Armour, 2020). Also in January 2020, the National Security Council received intelligence reports arguing that substantial spread of the virus would begin in the U.S. soon and Assistant to the President Peter Navarro issued a memo on January 29 warning “of half a million deaths and trillions of dollars in economic losses” (Lipton et al., 2020). By January 31, Secretary Azar had declared a public health emergency and Trump limited travel to and from China. It appeared at this stage that the gears of a governmental response to COVID were starting to turn, but the public was not made aware of the level of threat for what was to come.

CDC also started its work on developing a test for the COVID-19 virus. Typically, CDC develops its own tests for novel pathogens because it often can act more quickly than the private sector. This was the case for both H1N1 and Ebola. In the first week of February, CDC tests began shipping, but it was quickly apparent that they did not work due to contamination of the negative controls (Patel, 2020). Because of the CDC’s testing failures, the U.S. fell rapidly behind the rest of the world in testing its population. Initially, the FDA barred state and private sector labs from developing and processing their own tests, but reversed course on February 29. While doing so allowed the CDC to leverage the diverse array of actors who could help develop a functional test, the time lag in testing was costly in getting the virus’s spread under control.

Political control of CDC from the White House began to filter into the numerous decisions that CDC had to make regarding its COVID mitigation recommendations. Senior CDC scientist Dr. Nancy Messonnier was sidelined after making comments on February 25, 2020 about the imminent threat of COVID-19 (Weiland, 2020). The President went so far as to threaten to fire Messonnier in a call to Azar and the White House began to exert more control over CDC messaging (Ballhaus & Armour, 2020). In developing its social distancing guidelines, Director Redfield was forced to negotiate with White House Budget director Russell Vought over restaurant-specific guidelines. Instead of explicitly recommending 6 ft. of distancing, CDC simply recommended distancing for restaurants as a result (Weiland, 2020). Vought, advisor to the president Kellyanne Conway, and the president’s daughter Ivanka Trump all weighed in on specific guidance. Conway pushed for houses of worship to be deemed an essential service and Trump was involved in advice for schools (Ballhaus & Armour, 2020; Weiland, 2020).

As the pandemic response wore on, Director Redfield became the target of White House ire. He was criticized publicly by the President after testifying before Congress regarding the severity of the pandemic. Azar and Michael Caputo, the assistant secretary for public affairs at DHHS, both castigated Redfield at different times over CDC reporting (Ballhaus & Armour, 2020). Chief of Staff Mark Meadows even argued against increasing funds for CDC and instead asked for $300 million in CDC funding to be shifted to NIH for a vaccine campaign. The President also ordered hospitals to send COVID data to DHHS instead of CDC, centralizing control of official data in Washington (The Editors, 2020). CDC tried to get around the White House by issuing updates to its existing guidance instead of new guidance, which would not require White House review, but it was asked to even send those updates for review (Weiland, 2020). CDC developed its own symptom tracker tool, but the Trump Administration negotiated with Apple to develop a similar tool and then ordered CDC to remove the tool from its website (Weiland, 2020). Finally, and perhaps most distressingly for scientists, the White House begin asking for revisions to CDC’s Morbidity and Mortality Weekly Report, a vital source of public health information.

Approaching 200,000 deaths, fatigue from spring and summer lockdowns, a coming Presidential election, and fears of a fall/winter surge in cases, the CDC became “bolder” in the fall of 2020 by issuing guidance without White House review and resuming press briefings by scientists (Weiland, 2020). This is reminiscent of Wood’s (1988) observations of bureaucratic elasticity in the Environmental Protection Agency (EPA) during the Reagan Administration. EPA workers initially reduced activity after initial appointment of a hostile Administrator and budget cuts. The volume of monitoring and abatement activity rebounded, however. The resignation of the first Administrator was an important inflection point in that activity. In the case of COVID, CDC began to recover its direct communication practices after states began opening and the White House turned its attention to the 2020 election.

The replacement of Director Redford with Rochelle Walensky by the incoming Biden Administration was not a panacea, however, for the CDC’s messaging problems. Granted, direct political interference appears to have abated, but the CDC was widely criticized for first being slow to recommend changes in masking policies for the vaccinated before rapidly changing its recommendations without warning (Thorp, 2021). Holden Thorp (2021), Editor-in-Chief of *Science*, argues that the CDC’s troubles reflect the challenges of reconciling its dual scientific and political/communication aims. Science often appears to the public to be lurching—drawing one conclusion then the opposite as new research emerges. Public messaging, however, needs to be consistent and prescriptive. Additionally, government officials must often with uncertain information. The nuance of science speak is not well suited for decisive decision making and clear public messaging. Thorp asks a critical question: “is the CDC director a scientist or a political leader?” (1017). Such questions have led doctors to call for making CDC an independent agency, not subject to DHHS control (Shah & Forman, 2020). Internally, employees at the CDC have argued the agency’s problems stem from a lack of funding, authority, and an internal culture shaped by both (Interlandi, 2021).

**Figure 2.**
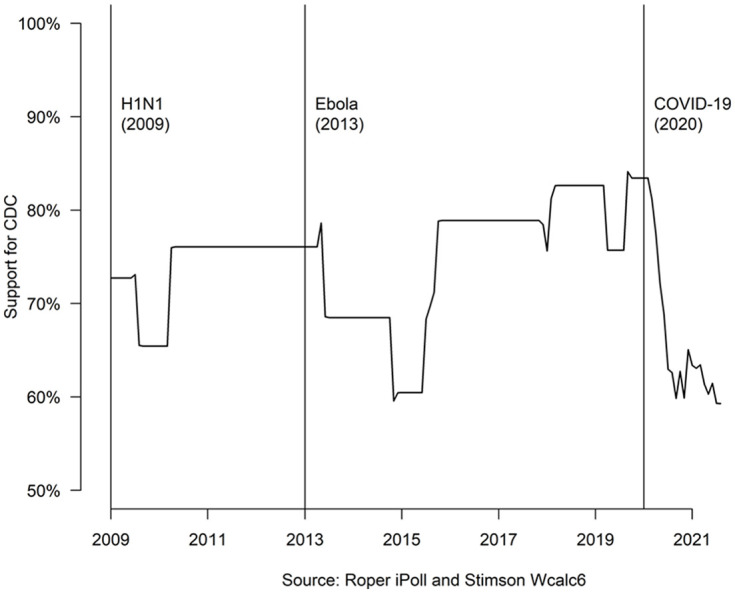
Aggregate public opinion in support of the CDC, 2009 to 2021.

**Figure 3.**
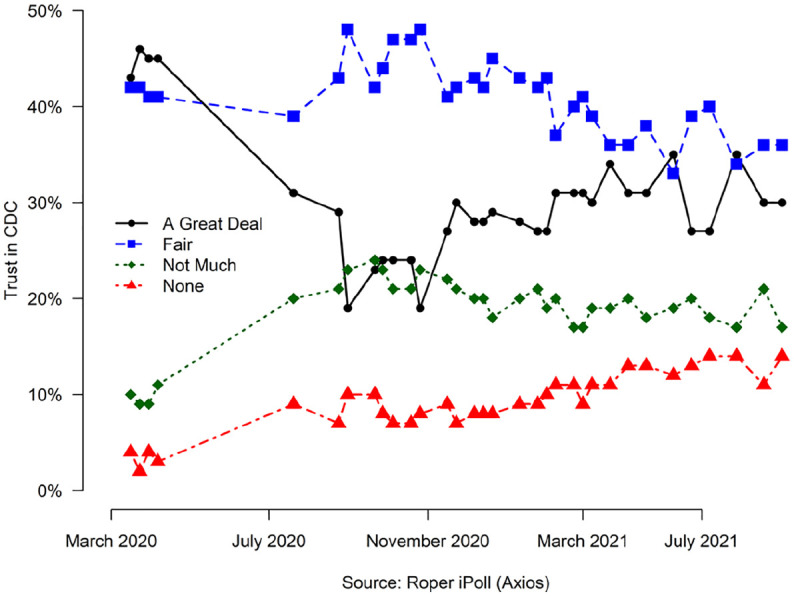
Axios poll of trust for CDC during the COVID-19 pandemic.

CDC’s reputation has undoubtably been challenged during the COVID-19 pandemic. Figure 2 aggregates national polling data from all polls in iRoper that asked a variant of the question of whether respondents trust/support the CDC from 2009 to 2021.^2^ There are notable dips in public trust during all three of our cases, but the deepest were during Ebola and COVID-19. It is also notable that trust in CDC was growing prior to the current pandemic. While support never falls below 50% in Figure 2, the relative strength of public opinion is not clear. Figure 3 offers another look at opinion by plotting responses to Axios’s periodic poll, which asked identical questions about trust in CDC during the COVID-19 pandemic. “A fair amount of trust” has slowly eroded, whereas “a great deal of trust” dropped precipitously and has been recovering slowly. ‘No trust’ in the CDC started close to 0% and increased to 13% by August 2021. Public confidence in CDC has clearly been undermined during the COVID-19 pandemic and remains low well past the end of the Trump Administration.

## Discussion

In comparing the three case studies, the CDC acted with stronger autonomy during Ebola and H1N1 than during COVID-19. It handled both the H1N1 and Ebola crises with excellence and largely retained the public’s confidence, with a rise of fear and mistrust more prevalent during Ebola. The same is not true for COVID-19. As we have argued, during COVID-19, not only did the CDC suffer from political interference from the Trump White House in terms of decision making and information dissemination, but it also suffered from bureaucratic failure beyond the malignancy of political interference.

Bureaucratic failure stems from institutional rigidity, diminishing institutional capacity, and political neglect from the Congress. Institutional rigidity is the inability of an agency to respond quickly or think entrepreneurially when a new situation, such as COVID-19 arises (Fukuyama 2014). While “some of [CDC’s] many pockets are bursting with innovation; others are plagued by inertia” (Interlandi, 2021). The CDC could not provide adequate guidance to school districts on how to keep schools open, or if they should close—forcing local districts to make individual plans tailored to their communities. Agency departments face a siloed culture among offices, which makes it more difficult to work across the agency and focus horizontally instead of vertically. Consequently, the CDC was slow to act. Staff scientists are often too reliant on published academic literature, which can prevent an entrepreneurial spirit in the face of a novel virus (Interlandi, 2021). The fact that the CDC failed to work effectively with the FDA during the first weeks of the crisis or enable university labs to process samples displays a lack of flexible thinking. Institutional rigidity is also affected by institutional inertia—the problem of being reactive instead of pro-active and taking a long-term view of crisis response. The agency, founded in 1946, is resistant to change and slow to modernize surveillance systems and data-sharing agreements with states (Interlandi, 2021). This rigidity hobbled the CDC’s response to, arguably, the largest public health crisis the U.S. has faced since the agency’s inception.

Bureaucratic failure is also a result of inadequate institutional capacity and lack of mission (Wolf, 1993). The CDC has the responsibility to safeguard American public health; however, it has no authority over state departments of health to enforce lockdowns, mask mandates, or disease surveillance and testing. In fact, American federalism challenged the nation’s response to the crisis (Kettl, 2020). The CDC does not have the authority or the mission to close airports or cease international travel from the world’s hot spots—or insist that international travelers be tested or quarantined upon entering the U.S.. If, however, the CDC were working with an effective president, this lack of authority could be mitigated, as occurred during H1N1 and Ebola; but, as already discussed, Trump exacerbated an already difficult situation.

Lastly, in addition to the internal bureaucratic failures that the CDC experienced, the agency has been, for years, neglected by Congress, which also contributed to the COVID-19 crisis. Politicians have known for decades that the budgeting process at CDC must be overhauled to provide flexibility to the agency, but there has been a lack political will in Congress. Although it has a multi-billion-dollar budget that has increased significantly over the past 20 years, CDC has 200 separate line items that cannot be consolidated or shifted to other purposes should the need arise (Interlandi, 2021). This means that CDC relies on increases in funding during public health emergencies, a tradition that was not followed during COVID-19. Its splintered budget and pet programs are fiercely defended to members of Congress by entrenched advocacy groups, leading to interest group politics. Congress has been aware of these problems for decades but has not consolidated funding streams for flexibility in shifting monies to other areas as priorities change. In short, the CDC’s failure during COVID stemmed from political interreference from the White House, internal bureaucratic failure, and neglect from Congress over decades.

The balkanized media and political polarization among news consumers have also made the CDC’s job harder. CDC’s public health messaging was already weak in its ability to break through a fragmented media landscape in 2013 during Ebola, but it suffered even more in 2021. Not only is its message filtered through pundits, journalists, late night hosts, and a bevy of social media outlets, but also now through highly ideological outlets like Newsmax, One America News Network, and Fox News (Motta et al., 2020). This means that not only is its message potentially distorted, ridiculed, or misinterpreted, there is greater opportunity for outright manipulation and misinformation. Unfortunately, misinformation has been shown to spread far more easily and quickly than real information online (Vosoughi et al., 2018), a trend that was borne out during COVID (Obiała et al., 2021). There is no simple solution to this problem, but the challenge will only continue to grow for CDC.

Granted, the CDC is not the only public health agency that struggled in the face of the COVID-19 pandemic. Countries varied greatly in the extent to which they over- or under-responded COVID cases and deaths (Shafi & Mallinson, 2022). The United Kingdom, and its Health Security Agency (HSA), has faced significant difficulty in keeping COVID-19 in check. Its experience of waves of infection tended to directly precede those in the United States. The National Health Service also famously, and embarrassingly, was undercounting COVID cases because of Public Health England’s reliance on Microsoft Excel (Kelion, 2020). But what makes CDC particularly important is its global status as the crown jewel of public health. And other democratic nations, such as South Korea, New Zealand, and Israel, were far more successful in responding to the crisis.

## Conclusion

Why did the U.S., the richest and most prepared nation on Earth for a pandemic, fail so spectacularly in its response to COVID-19, especially compared to other countries with fewer resources? The CDC, the world’s “premier public health agency,” (McCay, 2020), did not meet the public’s expectations to limit the spread of the disease. In a perfect storm, the CDC was crippled with political interference from the Trump Administration and internal bureaucratic rigidity that stifled the dynamism necessary to address this novel coronavirus.

In the three case studies we examined, H1N1, Ebola, and COVID-19, the CDC performed meritoriously in two instances, and poorly in the third. What could have been done differently to avert this disaster and the deaths of over 700,000 Americans (as of October 2021)? There are many policy prescriptions that Congress and President Biden should consider. Though there is a long list, we offer three ideas. First, while the CDC enjoys a lot of bureaucratic autonomy, it lacks bureaucratic *authority* both at the federal and state levels due to the U.S.’ federalist structure. CDC needs greater authority over state departments of health and enforcement power. Providing a coordinated yet locally nuanced approach to mask mandates, lockdowns, and the like would have helped enormously in 2020. The CDC director should also be a Senate confirmed position, which would add to its authority and prestige (Florko, 2020). This would encourage better Congressional relations with the agency. Second, the CDC needs a major reorganization of both its siloed offices and its sprawling budget, giving the director more flexibility to divert resources where they are most needed. Enabling bureaucratic entrepreneurship in the agency and combatting inertia and intransigence would lead to a more pliable and nimble organization. Finally, in times of great crisis, the CDC must have the authority to recommend that travel from certain hot spots be curtailed, that overseas visitors must quarantine, and that testing is widely available. Presently this requires coordination with DHS, and thus the blessing of the President. Greater autonomy is required for CDC to behave nimbly in the face of emerging public health emergencies. The CDC stopped the spread of malaria in the U.S., and in the 21st century, Americans need a public health care agency that can flexibly adapt to novel situations.

## Notes

1. There is an extensive theoretical literature on principal-agent problems as applied to government that for space concerns we cannot fully discuss here. For more information on principal-agent theory see Kiewiet and McCubbins (1991), Lowery and Marchetti (2012), and Miller (2005).

2. Aggregate opinion is calculated using Stimson’s (1999) Wcalc6.
